# RETRACTION: Bee Venom Triggers Autophagy-Induced Apoptosis in Human Lung Cancer Cells via the mTOR Signaling Pathway

**DOI:** 10.1155/jo/9808753

**Published:** 2025-11-14

**Authors:** Journal of Oncology

RETRACTION: J. E. Yu, Y. Kim, D. E. Hong, et al., “Bee Venom Triggers Autophagy-Induced Apoptosis in Human Lung Cancer Cells via the mTOR Signaling Pathway,” *Journal of Oncology*, no. 2022 (2022), https://doi.org/10.1155/2022/8916464.

The above article, published online on 23 December 2022 in Wiley Online Library (https://wileyonlinelibrary.com), has been retracted by John Wiley & Sons Ltd.

The retraction has been agreed following an investigation of the concerns raised by *Actinopolyspora biskrensis* on PubPeer [1], which identified multiple instances of inappropriately overlapping figures as follows:

- In Figure 1(d), the 48-h panel appears identical to the snake venom + HCQ panel of Figure 2(b) when rotated, while describing different experiments.
- In Figure 2(b), all panels are unexpectedly similar to the images shown in Figure 2(b) of [2], where they describe results of a different experiment.
- In Figure 4(b), the Western Blot bands corresponding to β-actin share similar features with the β-actin bands shown in Figure 6(g) of [3], which were obtained using different experimental conditions.

As a result of our investigation, the conclusions of the article can no longer be considered reliable, and it is therefore retracted.

The authors have been informed of this retraction but did not provide a response.
